# Non-Heart-Beating Donor: An Extraordinary Example of Translational Medicine Research

**Published:** 2012-10-11

**Authors:** Rosalba Romano

**Affiliations:** Department of Medicine, Salerno University

**Keywords:** NHBD, HBD, Transplantation, Translational medicine

The definition of translational medicine integrates research from clinical and basic, social and political sciences, where the most important process is the conversion of biological discoveries into medical advantages.

According to Marincola, translational medicine “is a two-way street where the drive to cure should be complemented by the pursuit to understand human diseases and their complexities” [1].

Transplantation from non-heart-beating donors (NHBD) is probably the most unusual example of translational medicine application, where the concept “Bench to Bedside and Bedside to Bench” has been liberally (and involuntarily) explicated.

Historically, transplantations began in 1668 when a physician called Rob van Meekeren used a fragment from a dog’s skull to cure a soldier’s lesion. It was the first of an interminable list of attempts. For the first successful transplants, we need to wait until 1963, when Thomas Starzl performed a series of liver transplants, but no patient survived more than one month. In the same year James Hardy achieved the first lung transplant. The first successful heart human transplant was, instead, performed in 1967 by Christian Barnard in South Africa. In all these transplants the donor was a NHBD, a cadaver whose death was declared after the interruption of cardiac activity [2]. Only after the definition of brain death by the “Ad Hoc Committee of the Harvard Medical School to Examine the Definition of Brain Death” in 1968, the history of transplantation began to be inextricably linked to the evolution of heart-beating donors (HBD) or brain death donors [2]. Thanks to the availability of HBDs, NHBDs were abandoned as source of organs for many years. Nowadays, donation after cardiac death is again a concept widely spreading among people to increase the amount of transplantable organs even if there is reluctance to accept NHBD transplantation, because of the fear of poorer quality of organs compared to HBD [3]. For years, in clinical practice, surgeons are retrieving again organs from patients whose death was declared after cardiac arrest and this has pushed forward the research in the field. At the moment, experimental studies using animals or cells and clinical investigations are performed at the same time, but first observations about NHBD transplantation derived from clinical studies and not from experiments on animals, as it could be expected. It was the clinical practise and the patients’ safety problems that brought forward the experimental observation on cells and animals: in this field the research went from the bedsides (of the deceased patients, unfortunately) to the experimental surgery benches. NHBD research focuses on grafts tolerance of ischemia, comparison of damage suffered after cardiac death and damage after brain death, and around improving grafts quality. The graft quality is strictly connected to the length of time of Warm Ischemia (WIT) and of Cold Ischemia (CIT). Every loss of time, every delay in the management of the donor and the graft is paid with a worse outcome of the organ and of the recipient. Organ transplants allow an effective treatment of patients with an end-stage organ failure that lead to an improvement in life quality and expectance. The necessity of an increased amount of transplantable organs makes the search of new organ sources essential. The knowledge of these sources of grafts is essential for the medical and paramedical staff, involved in death declaration and in the whole transplant proceedings since NHBD is not only a technical problem but an ethical and cultural one [4].
